# Obstetric and Perinatal Outcomes in Pregnant Women with Lupus: Retrospective Study in a Portuguese Tertiary Center

**DOI:** 10.1055/s-0043-1772481

**Published:** 2023-11-09

**Authors:** Inês Ferreira Jorge, Joana Mourão Vieitez Frade, Susana Paula Leonardo Dias Abreu Capela, André Laboreiro Ferreira Mendes da Graça, Maria Luísa Aleixo Gomes Pinto Grilo, Ana Mónica Miguel Mendonça de Castro Centeno

**Affiliations:** 1 Serviço de Ginecologia e Obstetrícia, Hospital Beatriz Ângelo, Loures, Portugal; 2 Serviço de Dermatologia, Centro Hospitalar Universitário de Lisboa Norte, Lisboa, Portugal; 3 Hospital de Santa Maria, Centro Hospitalar Universitário Lisboa Norte, Lisboa, Portugal; 4 Faculdade de Medicina da Universidade de Lisboa, Centro Académico de Medicina de Lisboa, Lisboa, Portugal

**Keywords:** lupus erythematosus, pregnancy, pregnancy outcome, neonatal systemic lupus erythematosus, Resumo, lúpus eritematoso, gravidez, resultado da gravidez, lúpus eritematoso sistêmico neonatal

## Abstract

**Objective** Pregnancy in women with lupus poses a higher risk of complications compared with the general population. The present study aimed to determine and describe the obstetric and neonatal outcomes of pregnant women with lupus.

**Materials and Methods** We conducted an observational retrospective study of pregnant women with the diagnosis of lupus, who were selected and followed at the Maternal-Fetal Medicine Clinic of our institution between January 2013 and July 2018. We analyzed 59 pregnancies and 52 newborns, and collected data regarding sociodemographic features, the preconception period, pregnancy, childbirth, postpartum and the newborn. A descriptive analysis of the variables was performed.

**Results** In 58% of the cases, the pregnancy was uneventful. We registered flares in 25% of the cases, preeclampsia in 3%, fetal growth restriction in 12%, gestational loss in 10%, preterm labor in 10%, postpartum complications in 20%, and small for gestational age newborns in 17% of the cases.

**Conclusions** Most pregnancies in women with lupus have favorable obstetric and neonatal outcomes. Prenatal counseling, adequate multidisciplinary surveillance, and optimized treatment of the disease are fundamental pillars for these good results.

## Resumo

**Objetivo** A gravidez em mulheres com lúpus representa um risco maior de complicações em comparação com a população em geral. O presente estudo teve como objetivo determinar e descrever os resultados obstétricos e neonatais de gestantes com lúpus.

**Materiais e Métodos** Realizamos um estudo retrospectivo observacional de gestantes com diagnóstico de lúpus, selecionadas e acompanhadas no Ambulatório de Medicina Materno-Fetal de nossa instituição entre janeiro de 2013 e julho de 2018. Analisamos 59 gestações e 52 recém-nascidos e coletamos dados referentes às características sociodemográficas, período pré-concepcional, gravidez, parto, pós-parto e nascimento. Foi realizada uma análise descritiva das variáveis.

**Resultados** Em 58% dos casos, a gravidez transcorreu sem intercorrências. Registramos surtos em 25% dos casos, pré-eclâmpsia em 3%, restrição do crescimento fetal em 12%, perda gestacional em 10%, trabalho de parto prematuro em 10%, complicações pós-parto em 20% e recém-nascidos pequenos para a idade gestacional em 17% dos casos.

**Conclusões** A maioria das gestações em mulheres com lúpus tem resultados obstétricos e neonatais favoráveis. Aconselhamento pré-natal, vigilância multidisciplinar adequada e tratamento otimizado da doença são pilares fundamentais para esses bons resultados.

## Introduction

Systemic lupus erythematosus (SLE) is a chronic, systemic, and immune-mediated disease that mostly affects women of childbearing age. 1

In the last years, there has been an increase in the overall survival rate, a higher number of pregnancies, and an improvement in obstetric and perinatal outcomes. This was due to greater access to preconception counseling and multidisciplinary surveillance throughout pregnancy, as well as better perinatal care. 2

However, this disease carries a significant risk of obstetric and perinatal complications, the pathogenesis of which is mainly related to uteroplacental insufficiency, the inflammatory state underlying the disease, and the possibility of maternal immunoglobulin G (IgG) autoantibodies crossing the placental circulation and binding to fetal tissues. 3 4

Regarding the obstetric complications, there is an increased risk of abortion, preterm birth, fetal death; the hypertensive complications include preeclampsia (PE), eclampsia (E) and/or hemolysis, elevated liver enzymes, low platelet count (HELLP) syndrome; other complications include gestational diabetes, fetal growth restriction (FGR), a higher rate of infections, thromboembolic complications, cesarean sections, and postpartum complications, including infection, hemorrhage, and lupus flares. 5 6 7

Many predictors of complications and adverse outcomes have been described in the literature, namely: lupus nephritis, damage to other organs (lung, heart, central nervous system), interruption of the medical treatment, active disease in the six months before conception, antiphospholipid syndrome (APS) or the presence of persistent antiphospholipid antibodies, hypocomplementemia, thrombocytopenia, and high levels of anti-double stranded DNA (anti-dsDNA), anti-Sjögren's-syndrome-related antigen A (anti-SSA/Ro), and anti-Sjögren's-syndrome-related antigen B (anti-SSB/La) antibodies. 8 9

Regarding newborns (NBs) of mothers with SLE, there is an increased risk of several complications, namely: prematurity, low birth weight. and neonatal lupus. 10 11 12

The present study aimed to assess the obstetric and perinatal outcomes of pregnant women with SLE.

## Materials and Methods

We conducted a retrospective observational study, in which we evaluated pregnant women diagnosed with SLE included in the database of the Materno-Fetal Medicine Clinic of our institution. We included women diagnosed with lupus who had been surveilled in the Obstetrics Department between January 2013 and July 2018. The study included 52 women, totaling 59 pregnancies and 52 NBs. We excluded pregnant women whose birth took place at another institution, as well as women and/or NBs whose clinical files were incomplete or unavailable for consultation.

We collected data regarding sociodemographic features and data relating to the pre-conception period, pregnancy, childbirth, postpartum and NB, and performed a descriptive analysis of the variables using Microsoft Excel 2013 (Microsoft Corp., Redmond, WA, United States) and IBM SPSS Statistics for Windows (IBM Corp., Armonk, NY, United States), version 25.0, with a confidence interval of 95% (95%CI) and a statistical significance level of 0.05. Early abortion was classified as a spontaneous pregnancy loss up to 11 weeks and 6 days; late abortion, as a pregnancy loss between 12 and 21 weeks and 6 days; fetal death, as an intrauterine death from 22 weeks onwards; and preterm delivery, as those occurring between 22 and 36 weeks + 6 days. Preeclampsia was defined by hypertension and proteinuria and/or organ dysfunction after 20 weeks, in a previously normotensive woman, and gestational hypertension referred to hypertension without proteinuria after 20 weeks, in a previously normotensive woman. Fetal growth restriction was defined through an ultrasound estimate of fetal weight below the 3rd percentile or an ultrasound estimate of fetal weight below the 10th percentile for gestational age along with Doppler changes. Small for gestational age (SGA) was defined as an estimate of fetal weight below the 10th percentile for gestational age without Doppler changes, and large for gestational age (LGA) referred to fetal weight above the 90th percentile for gestational age. We also defined flare as an increase in lupus activity in a patient with inactive disease, and postpartum complications, as hypertensive, hemorrhagic, infectious complications, and postpartum anemia.

The present study was approved by the Ethics Committee of the hospital where it was performed, and international ethical standards were followed.

## Results

**Fig. 1:**
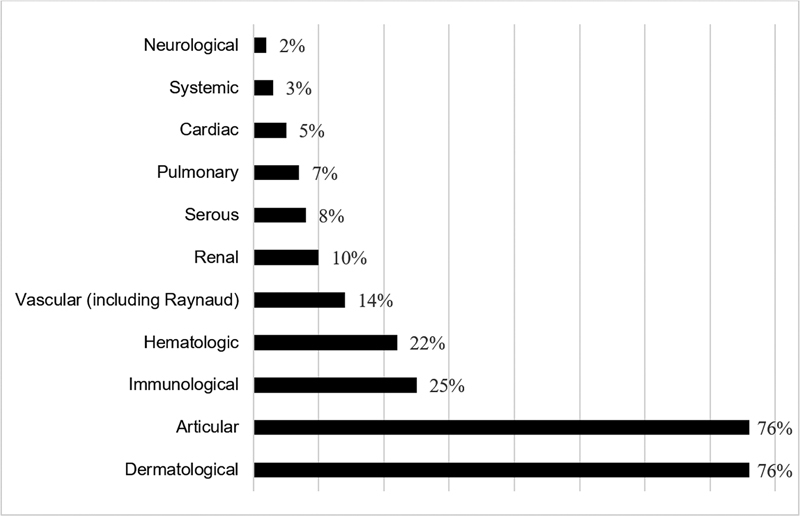
Preconceptional manifestations of lupus, relative frequency ( *n* = 59).

Starting with the demographic features ( Table 1 ), the mean age of the pregnant women was of 33.5 ± 5.6 (range: 18 to 46) years, 92% were Caucasian, and 8% were of African origin. Regarding parity and previous obstetric history, most women were multiparous (53%), and there was a history of prior abortion in 25% of the cases, half of which were associated with APS; in 7% of the cases, there was a history of fetal death, half of them associated with APS. As far as lupus is concerned, the mean duration of the disease at the beginning of the obstetric follow-up in the Materno-Fetal Medicine Clinic was of 10.0 ± 6.3 (range: 0 to 25) years. The most frequent lupus manifestations were cutaneous, articular, immunological, and hematological ( Fig. 1 ). We registered a thromboembolic history in 14% of the cases and other rheumatologic diseases in 10%.

**Table 1:** Sociodemographic, clinical, and laboratory features of the study sample

Sociodemographic, clinical and laboratory features	Frequency (n)	%
***Sociodemographic features***		
**Caucasian race**	54	92
**African origin**	5	8
**Age: 18–29 years**	18	30
**Age: 30–39 years**	34	58
**Age: 40–46 years**	7	12
**Nulliparous**	28	47
**Multiparous**	31	53
**Duration of the disease < 10 years**	24	41
**Duration of the disease ≥ 10 years**	35	59
***Clinical features***		
**Active disease in the preconception period**	6	10
**Lupus nephritis**	6	10
**Antiphospholipid syndrome**	15	25
**Hypertension**	6	10
**Secondary to lupus nephritis**	4	7
**Primary**	2	3
***Laboratory features – analytical alterations***		
**Anemia**	8	14
**Leu** k **openia**	3	5
Lymphopenia	3	5
**Neutropenia**	1	2
**Thrombocytopenia**	4	7
**Hypergammaglobulinemia**	1	2
**Hypocomplementemia**	8	14
**Proteinuria**	10	17
Not applicable*	3	5
***Laboratory features – antibodies***		
**Antiphospholipid antibodies**	17	29
**Anti-** ribonucleoprotein	9	15
**A** ntinuclear antibodies	37	63
**Anti-** double stranded **DNA**	15	25
**Anti-** Sjögren's-syndrome-related antigen A	18	31
**Anti** -Sjögren's-syndrome-related antigen B	8	14
Not applicable*	3	5

**Note:** *Not applicable due to early abortion, and no analytical evaluation was performed ( *n* = 59).

Before getting pregnant, most women in the sample (76%) did not undergo preconception consultation. Most were singleton pregnancies, but we registered one case of twin pregnancy. Regarding the clinical risk factors ( Table 1 ), most pregnancies started with the disease in an inactive state or in remission (90%). We observed lupus nephritis in the current pregnancy in 10% of the cases, APS in 25%, and hypertension in 10%, which was secondary to lupus nephritis in most cases (67%). Considering the laboratory findings ( Table 1 ), anemia occurred in 14% of the pregnancies, thrombocytopenia, in 7%, hypocomplementemia, in 14%, and proteinuria, in 17%. Regarding the antibody profile, there were positive antiphospholipid antibodies in 29% of the cases, positive anti-ribonucleoprotein (anti-RNP) antibodies in 15%, positive anti-dsDNA in 25%, and positive antinuclear antibodies (ANAs) in 63%, which were anti-SSA in 31% of the cases and anti-SSB in 14% of te cases, the latter always occurring concomitantly with the presence of anti-SSA. We registered some complications of the pregnancy ( Table 2 ), namely hypertensive complications, flares, growing disturbances, and abortive outcome; hypertensive complications were recorded in 10% of the cases, with 3% corresponding to PE and 7%, to gestational hypertension; 25% of the cases had a lupus flare, with the most frequent manifestations being skin rash, joint pain, vasculitis, and worsening of the proteinuria; FGR occurred in 12% of the pregnancies, and we recorded early abortion in 5% of the cases, late abortion in 3%, and fetal death in 2% ( *n* = 1). The case of fetal death occurred at 30 weeks, in a fetus with trisomy 18. Delivery was uneventful in most cases. The mean gestational age at delivery was of 38 weeks and 4 days ± 10 days, with preterm delivery occurring in 10% of the cases. We registered normal vaginal delivery in 32% of the cases, delivery assisted by vacuum extraction in 9%, forceps in 9%, forceps after failed vacuum extraction in 6%, and cesarean section in 44% of the cases (planned in 25% and intrapartum in 19% of the cases). The immediate postpartum period was marked by complications in 20% of the cases ( Table 3 ): de novo hypertension in 2%, anemia in 10%, postpartum hemorrhage in 2%, and infection in 7%. We did not observe thromboembolic complications. In the postpartum period, we recorded flares in 2% of the cases, with hematological manifestations.

**Table 2:** Complications during pregnancy and the postpartum period ( *n* = 59)

	Frequency (n)	%
**Complications of pregnancy**		
**Fetal growth restriction**	7	12
**Gestational diabetes**	6	10
**Gestational hypertension**	4	7
**Early abortion**	3	5
**Preeclampsia**	2	3
**Late abortion**	2	3
**Respiratory infection**	2	3
**Pregnancy cholestasis**	2	3
**Thromboembolic events**	1	2
**Fetal death**	1	2
**Complications of the postpartum period**		
**Hypertensive (de novo hypertension)**	1	2
**Anemia**	6	10
**Hemorrhage**	1	2
**Infection**	4	7
*** Respiratory***	2	3
*** Urinary***	1	2
*** Surgical wound***	1	2

**Table 3:** Therapy administered in the preconception period, throughout pregnancy, and the postpartum period ( *n* = 59)

Treatment	Preconception: n (%)	Pregnancy: n (%)	Postpartum: n (%)
**None**	11 (19%)	4 (7%)	4 (7%)
**Hydroxychloroquine**	47 (80%)	50 (85%)	50 (85%)
**Prednisolone**	33 (56%)	33 (56%)	34 (58%)
**Azathioprine**	13 (22%)	15 (25%)	15 (25%)
**Tacrolimus**	0	2 (3%)	2 (3%)
**Acetylsalicylic acid**	23 (39%)	34 (58%)	34 (58%)
**Enoxaparin**	10 (17%)	19 (32%)	18 (31%)

As far as treatment is concerned ( Table 3 ), 19% of the pregnancies occurred in women who were not undergoing any type of treatment. In total, 80% of the medicated women were taking hydroxychloroquine (HCQ), 56% were under corticosteroid therapy with prednisolone, 22% were medicated with azathioprine, 39% were taking acetylsalicylic acid (ASA), and 17%, enoxaparin. During pregnancy, most women were medicated with HCQ (85%) and/or prednisolone (56%), and 25%, with azathioprine, and 3% with tacrolimus. Most pregnant women took ASA (58%), and 32% took enoxaparin. In the postpartum period, therapy was similar to that recorded during pregnancy.

We had a sample of 52 NBs, and 59% of them were female. The mean birth weight was of 2,968 ± 462 g (range: 1,920 g to 3,845 g), with 17% of the cases being SGA. We did not register any LGA NBs. In 90% of the cases, the NBs were hospitalized with their mothers, and 10% needed special neonatal care. Most NBs had no clinical or laboratory alterations. Upon physical examination ( Table 4 ), 31% had jaundice, 2% had exanthema, and 8% had alterations in the primitive reflexes; there were no other relevant changes. Regarding the NBs who underwent analytical evaluation ( Table 4 ), 5% had anemia, 2% had thrombocytopenia, 48% had hyperbilirubinemia, 5% had an increase in gamma-glutamyl-transferase, and 13% had hypoglycemia; the autoimmune profile was determined in a minority of NBs. An electrocardiogram (ECG) was requested in 71% of the NBs, with no alterations in the atrioventricular conduction.

**Table 4:** Alterations on the physical and laboratory examinations of the newborns

	Frequency (n)	%
**Physical examination**		
Exanthema	1	2 ^a^
Jaundice	16	31 ^a^
Heart murmurs	1	2 ^a^
Respiratory distress syndrome	1	2 ^a^
Primitive reflex changes	4	8 ^a^
**Laboratory findings**		
Blood count	43	83 ^a^
* Anemia*	2	5 ^b^
* Leucopenia*	8	19 ^b^
Platelet count	42	81 ^a^
* Thrombocytopenia*	1	2 ^c^
Liver profile	21	40 ^a^
* Increased transaminases*	3	14 ^d^
* Hyperbilirubinemia*	10	48 ^d^
* GGT increase*	1	5 ^d^
Antibody profile	5	10 ^a^
* Positive antibodies*	3	60 ^e^
* Antinuclear antibodies*	3	75 ^f^
* Anti-double stranded DNA*	1	50 ^c^
* Anti-Sjögren's-syndrome-related antigen A*	1	20 ^e^
* Anti-Sjögren's-syndrome-related antigen B*	1	20 ^e^
Complement dosage	1	2 ^a^
* Hypocomplementemia*	1	100 ^g^
Blood glucose measurement	23	44 ^a^
* Hypoglycemia*	3	13 ^h^

**Notes:**^a^ n = 52; ^b^ n = 43; ^c^ n = 2; ^d^ n = 21; ^e^ n = 5; ^f^ n = 4; ^g^ n = 1; ^h^ n = 23.

## Discussion

In the series herein presented, the percentage of pregnancy loss was of 8%. This value is below the average found in the literature, with values in the order of 20% to 30%, probably related to the lack of records of early abortions. 12 13 There are some identified risk factors for abortion, namely: a history of abortion, proteinuria, APS, AAF, thrombocytopenia, hypocomplementemia, positive anti-dsDNA antibodies, HTA, exacerbations, previous lupus nephritis, PE/E, active disease in the six months before conception, and inaugural SLE in pregnancy. 7 8 14 15 16

Of the 5 cases of women with abortion that we recorded, 4 had concomitant APS, and this condition was registered in 25% of the study sample. The association between APS and adverse obstetric outcomes has been described, including miscarriages, FGR, PPT, and hypertensive disorders in pregnancy. 8 14 15 16

Recent data report an European rate of prematurity ranging from 5% to 9%, which contrasts with the 30% to 33% reported in the literature regarding the global population of NBs whose mothers have SLE. 17 18 19 In the sample of the present study, we found 10% of preterm deliveries, a percentage substantially lower than the values reported in the literature concerning the global population with SLE. We highlight that all the preterm NBs in the present series were late preterm.

Several studies 5 7 8 14 20 21 report an association of preterm delivery with some risk factors, namely: high disease activity at conception, positive antiphospholipid antibodies, APS, flares, obstetric history of abortion, thromboembolic complications, previous lupus nephritis, hypertension, PE/E, hypocomplementemia, proteinuria, positive anti-dsDNA antibodies, thyroid disease, and prednisolone treatment dose higher than 15 mg/day. 5 7 8 14 20 21

Fetal growth restriction occurred in 12% of the cases, which is similar to the values already described, which range from 6% to 30%. 5 12 13 22 The literature describes a relationship between FGR and some predictors, namely: APS, positive antiphospholipid antibodies, hypertension, or lupus nephritis. 9

As for PE, it occurred in 2 cases (3%), 1 of which overlapped with chronic hypertension. This percentage is in the range of incidence referred in different studies, which is of 3% to 30%, and is also similar to the percentage found in the population of pregnant women without SLE (∼ 5%). 5 12 13 18 22 23 There is a well-described relationship between PE and the existence of lupus nephritis, 14 24 as well as with some other risk factors, such as positive antiphospholipid antibodies, APS, hypocomplementemia, and positive anti-RNP or anti-dsDNA antibodies. 5 8 18 24 25 26

In about a quarter of the pregnancies (25%), SLE flares occurred, the most frequent manifestations being cutaneous and articular, which is in agreement with the literature. 9 24

It is known that active disease in the preconception period is a predictor of the occurrence of flares. In the present study, it is possible that the absence of disease activity before pregnancy, which was observed in most patients (90%), contributed to the low rate of flares (∼ 25%), which is comparable to the rates reported in other studies, with an incidence ranging from 10% to 33%. 18 27 28

Most of the sample of the present study had a mild form of the disease, which was probably the main contributing factor to the considerably favorable results. On the other hand, most patients (85%) received HCQ as part of their treatment during pregnancy, which may have also contributed to the good outcomes obtained.

Hydroxychloroquine was identified as a protective factor against adverse outcomes, especially flares. 29 The literature also associates this drug with possible preventive effects regarding to congenital atrioventricular block, and some studies also report a therapeutic effect on this condition, as well as effects in the reduction if prematurity, FGR and SGA. 8 30 31 In the present study, we did not identify changes in atrioventricular conduction in any of the NBs in the sample.

Flares in the immediate postpartum period were much less significant, with only one case, which occurred in a patient with a record of exacerbation during pregnancy. Some studies have demonstrated that patients with flares during pregnancy have an increased risk of developing postpartum exacerbations. 18

The rate of SGA NBs in the general population is of ∼ 10%. 19 In the present study, we found that 17% of the NBs were SGA, which is in agreement with the results obtained in previous studies (10% to 30%). 10 There are variables associated with SGA NBs, including African origin, prematurity, hypertensive complications or lupus nephritis. 24 26 32

Neonatal lupus is a rare syndrome, which occurs in 1% to 2% of NBs to mothers with anti-SSA and/or anti-SSB autoantibodies, manifesting more frequently by cardiac, cutaneous, hematological, and hepatic alterations. In the present study, none of the documented alterations in the NB was presumably associated with an autoimmune etiology; as an example, the high incidence of jaundice can be related to hemolytic disease of the NB. However, it should be noted that the manifestations of neonatal lupus can mimic many other neonatal pathologies, possibly appearing at a later stage.

The percentage of NBs in need of special neonatal care was similar to the percentage of general NBs in our institution with this need (10% versus 11%).

The present study incorporates the limitations inherent to a small sample resulting from a retrospective analysis of a single center and for a limited period of time. Other limitations are related to the exclusive access to the records of the NBs during the period of hospitalization, and we did not consult records related to subsequent hospitalizations, follow-up appointments or admissions to the emergency service, which would be of interest, given that the manifestations related to neonatal complications in NBs to mothers with SLE may appear during the first weeks/months of life. 4 The fact that pregnant women who gave birth outside our institution were not included led to the exclusion of part of the pregnancies in follow-up, which constitutes another limitation of the present study, as well as the fact that the absence of electronic clinical records during the study period led to the exclusion of pregnant women and NBs whose handwritten files were not available for consultation or had incomplete data.

## Conclusion

In conclusion, we found that most women in this sample had good obstetric and neonatal outcomes, with low rates of abortion, preterm birth, and PE. The most frequent complications were FGR and SGA NBs, but even so, with rates similar to those obtained in other series. The fact that most women had a mild form of the disease and were taking HCQ during pregnancy may have contributed to the good obstetric and neonatal outcomes observed. Thus, although most pregnant women with SLE have favorable obstetric and perinatal outcomes, these women continue to represent a risk group for obstetric complications. To improve obstetric and perinatal outcomes, it is essential to plan the pregnancy during a remission phase of the disease, so that adequate multidisciplinary surveillance of the pregnancy and optimal treatment of the disease can be performed, as well as to plan the delivery in a differentiated perinatal center.
